# Association between overtime and depressive symptoms among Chinese employees

**DOI:** 10.3389/fpubh.2023.1241994

**Published:** 2023-10-11

**Authors:** Yinyin Liang, Zixuan Li, Xinrou Wang, Pengcheng Liu, Liang Ma, Xiaojie Wang

**Affiliations:** ^1^Nottingham University Business School China, University of Nottingham Ningbo China, Ningbo, China; ^2^School of Business, University of Leeds, Leeds, United Kingdom; ^3^School of Economics, Qingdao University, Qingdao, China; ^4^Department of Child Health, Affiliated Hospital of Qingdao University, Qingdao, China; ^5^School of Management, Ocean University of China, Qingdao, China

**Keywords:** overtime, heavy overtime, depressive symptoms, mental health, age, education level, income level

## Abstract

**Objectives:**

This study examines the correlation between overtime and depressive symptoms, and analyzed the moderating effect of age, education level, and income level on their correlation by using a nationally representative sample from the Chinese Family Panel Studies (CFPS) in 2018.

**Methods:**

Participants are divided into three groups: 30-44 h/week, 44.1–61.9 h/week (defined as overtime group), and ≥ 62 h/week (heavy overtime group). The multiple ordered logistic regression models are conducted to estimate the association between overtime and depressive symptoms. The interaction term of overtime and moderators including age, education level, and income level are introduced into the models to test the moderating effect.

**Results:**

The overtime group have an increased probability of depressive symptoms (OR = 1.11, 95% CI 1.04–1.20) compared with those who reported working hours 30-44 h/week, after controlling for important confounders. What’s more, the worsening of depressive symptoms is more pronounced in the heavy overtime group (OR = 1.32, 95% CI 1.22–1.44). The moderating effect results show that younger employees, employees with high education levels, and those with high income level are more likely to be affected by the negative effects of overtime.

**Conclusion:**

Working overtime increased the likelihood of depressive symptoms. Younger employees, high-educated employees and employees with high income level are more vulnerable to the negative effects of overtime on mental health.

## 1. Introduction

The right to rest is a necessary requirement for employees’ subsistence rights. However, the phenomenon of forcing employees to work overtime is relatively common and long-standing in China. As for employees, long-term overtime work may not only cause physical health problems such as headaches, myocardial infarction and stroke (1, 2), but also harm their mental health by increasing the risk of anxiety, depressive symptoms, and even depression (3).

Depressive symptoms are the early manifestations of depression, which are more prevalent in the population. Most of the existing studies prove that overtime significantly elevates the probability of depressive symptoms occurring (4–6). However, there are a few studies that point out that for specific groups, overtime has no significant effect on depressive symptoms. For example, Virtanen et al. (3) noted that long working hours were a risk factor for depression and anxiety symptoms in women, but the effect was not significant for men. Nakata (7) stated that long working hours were associated with an increased risk of depressive symptoms only in the context of reduced job satisfaction. The inconsistency of the findings may be due, on the one hand, to differences in the measurement of the extent of overtime work. There are significant country-specific differences in the extent of overtime due to different levels of development and overtime cultures (8, 9). Compared to developed countries represented by the United States and the European Union, Asian countries such as China tend to gain a relative development advantage over overtime, resulting in a prevalent overtime culture due to the relative backwardness of industrial development and the serious homogenization of international competition. Consequently, empirical evidence from non-Western societies such as China would more accurately demonstrate the relationship between overtime and mental health (4). On the other hand, inconsistency in findings may stem from variability in the characteristics of the study population. For example, background or boundary factors such as socioeconomic background, demographic characteristics, and individual job characteristics may moderate the association between overtime and depressive symptoms. However, few studies have revealed the conditioning factors that influence this association.

Motivated by the research gaps, this study analyzes the association between overtime and depressive symptoms using data of CFPS2018. The results reveal that overtime increased the likelihood of depressive symptoms after controlling for individual characteristics variables, health status variables, and health behavior variables. The measurement results of moderating effects show that young employees, well-educated employees and high-income employees are more likely to be affected by the negative effects of overtime. To some extent, the conclusions of this study can explain the potential controversy in academic circles about the effect of overtime on depressive symptoms, and provide a reference for public health departments to prevent and intervene with depressive symptoms among employees.

The marginal contributions of this paper are as follows. Firstly, compared with Western countries where overtime is relatively less severe, overtime in China is more typical. This paper provide empirical evidence from China for studying the link between overtime and depressive symptoms. Secondly, three moderating variables of age, education level, and income level are introduced into the empirical framework of the influence of overtime on depressive symptoms, which expanded the analysis perspective of the influencing factors of depressive symptoms. Especially, most of the existing studies introduce age as a control variable but ignored the moderating effect of age on the relationship between overtime and depressive symptoms. Thirdly, contrary to the conclusion of mainstream research that education level and income level negatively regulate depressive symptoms, this study finds that these two variables positively moderate the relationship between overtime and depressive symptoms in China based on CFPS2018. The reason may be that the prevailing overtime culture makes the conclusions of other countries inapplicable to China.

## 2. Materials and methods

### 2.1. Data source and study sample

The data analyzed in this article are derived from the Chinese Family Panel Studies (CFPS), conducted by the Institute of Social Sciences (ISSS) of Peking University in 2018. Initiated, CPFS is a nationwide, large-scale, multidisciplinary social research project. The survey obtained data from three levels: individual, family, and community. The sample covers 25 provincial administrative units, with a target sample size of 16,000 households. All members of the sampled households over the age of 9 are interviewed. The survey investigates the economic and non-economic well-being of Chinese residents, which is widely used for academic research and public policy analysis. In the 2018 survey, the CFPS measured individuals’ depressive symptoms with an eight-item version of the Center for Epidemiological Studies-Depression (CES-D8) scale, which provides visible data support for this study.

**Figure 1:**
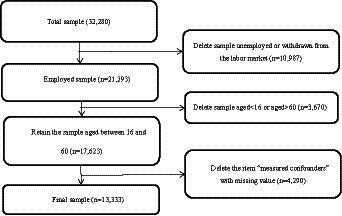
Study sample selection process.

In the 2018 survey, 32,280 individuals have reported depressive symptoms. Based on this, this paper processes the data as follows: those who are unemployed or withdrawn from the labor market (*n* = 10,987), not of working age (<16 years old or > 60 years old) (*n* = 3,670), and those with data missing (*n* = 4,290) are excluded from the analysis (Figure 1).

### 2.2. Measures

#### 2.2.1. Overtime

In the CFPS survey, the specific question regarding weekly work time is set as follows: “Weekly work time (hours) does not account for lunch breaks but includes overtime, regardless of whether it is paid or not. The inquiry then inquires about the average number of hours one has typically worked in this job over the past 12 months.” According to the earlier studies, overtime is measured by the total hours of additional work time excluding lunch breaks per week, regardless of whether paid or not (10, 11). The legal working hours in China are regulated to 44 h/week according to the *Labor Law of the People’s Republic of China*. Based on this, overtime in this paper is defined as work time more than 44 h/week. If it is necessary to extend working hours due to special reasons, the extension of working hours shall not exceed 3 h a day under the condition that the health of employees is guaranteed, meanwhile, the employees have at least one day off per week, which means that employees should not work more than 62 h a week. Therefore, we divide participants into three groups based on working hours per week: 30-44 h/week, 44.1–61.9 h/week, and ≥ 62 h/week. Workers who work 44.1–61.9 h/week are defined as overtime workers and ≥ 62 h/week as heavy overtime workers.

#### 2.2.2. Depressive symptoms

The eight-item short form of the Centre for Epidemiological Studies Depression Scale (CES-D8) is used to assess the level of depressive symptoms. The CES-D is widely used as a self-evaluation test in the general population (12). The Chinese version of the scale has been proven satisfactory in construct validity and psychometric properties in previous studies. This scale consists of 8 items assessing negative mood and somatic symptoms over the past week. Participants are asked six positive questions (e.g., felt lonely) and two reverse-scored questions (e.g., felt happy) to assess the frequency of the above symptoms (less than 1 day, 1–2 days, 3–4 days, or 5–7 days, assign values from 0 to 3). The total CESD-8 score can be calculated from 0 to 24, with higher scores indicating more severity of depressive symptoms.

#### 2.2.3. Health status

This study uses self-reported health status and chronic disease as measures of health status. Self-rated health is a sensitive and reliable indicator of an individual’s current overall physical health, which is recorded by the 5-point Likert scale for the question “How would you rate your health status?” The responses are classified into three levels (unhealthy, fair, and healthy). Chronic diseases are typically classified into several major categories, including cardiovascular diseases (like heart disease and stroke), nutritional and metabolic diseases (like obesity and diabetes), cancers, mental and psychological disorders (such as depression and anxiety disorders),chronic respiratory diseases (like chronic bronchitis and asthma) and connective tissue and rheumatism (13). And it is measured by whether the respondents have been suffering from doctor-diagnosed chronic conditions in the past 6 months (0 = no and 1 = yes).

#### 2.2.4. Health behaviors

Health behaviors are measured by four health factors including sleeping time weekday, physical exercise, smoking, and alcohol consumption in this study. Sleeping time weekday is analyzed with the question: “In general, how many hours of actual sleep do you spend on workday nights?” Physical exercise is dichotomized according to the intensity and frequency of physical activity during the past week (0 = no and 1 = yes). Smoking is classified by asking respondents: “Have you smoked over the past month?” (0 = no and 1 = yes). Alcohol consumption is determined by asking whether the individual had drunk alcohol more than three times a week in the past month (0 = no and 1 = yes).

#### 2.2.5. Socio-demographic characteristics

Demographic characteristics include age (18–60), gender, marital status (married, unmarried, divorced, or bereavement), and educational level (middle school and below, high school/vocational school, and college and above). In addition, this paper also introduces the subjective and objective indicators of an individual’s income level as control variables. Personal income as an objective indicator is calculated by the total personal income in the past 12 months.

### 2.3. Statistical analysis

Associations between overtime and socio-demographic variables are analyzed using Pearson’s chi-squared test for categorical variables and a t-test for continuous variables. The CES-D8 ranging from 0–24 records respondents’ depressive symptom scores from low to high. Given the ordered categorical nature of the depressive symptoms variable, ordered logistic regression is used to capture the qualitative differences between different depressive symptom levels (14, 15). We also tested the assumption of proportional odds for all models by employing the test of parallel lines. So the multiple ordered logistic regression models are conducted to estimate the association [odds ratio (OR) and 95% confidence intervals (CIs)] between overtime as the independent variable and depressive symptoms as the dependent variable. Each OR may be interpreted as the effect of the variable on the odds of a higher-category outcome for CES-D scores and depressive symptoms, higher being better. Those working 30–44 h/week are set as the reference group. In Model 0, the association between overtime and depressive symptoms is assessed after controlling for age, gender, marital status, education level, and self-evaluation income. In Model 1, health status indicators (self-rated health and chronic disease) are added based on Model 0. Health behaviors indicators (physical exercise, smoking, alcohol consumption, and sleeping time weekday) are added in Model 2. The moderating effects of age, education level, and income level are further examined to estimate the association [odds ratio (OR) and 95% confidence intervals (CIs)] between overtime and depressive symptoms, statistical significance is considered at *p* = 0.05. All analyses are performed using STATA version 14.0 (STATACorp, College Station, Texas).

## 3. Results

### 3.1. Characteristics of study population

The univariate analysis results according to participants’ weekly working hours are reported in Table 1. Among all participants, 4,161 (31.21%) are distributed in the non-overtime group who worked 30–44 h/week, 5,303 (39.77%) are distributed in the overtime group who worked 44.1–61.9 h/week, and the other 3,869 (29.02%) are distributed in heavy overtime group who worked ≥62 h/week. The mean (±standard deviation) age is 40.06 ± 11.07 years in the study population. Gender, marital status, education level, and personal income, self-rated health, chronic disease, sleeping time, physical exercise, smoking, and alcohol consumption are statistically significantly (*p* < 0.05) associated with weekly working hours. The results show that individuals who reported overtime are more likely to be male, married, less educated, with no chronic diseases, who exercised less, smoked, and drank (all *p* < 0.05).In addition, we also find that the income level of the heavy overtime group (working hours≥62 h/week) is lower than that of the overtime group (working 44.1–61.9 h/week).

**Table 1:** Baseline background characteristics of participants with and without overtime.

Variables	Total population 13,333 (100%)	Working hours/week			
		30–44 h/week	44.1–61.9 h/week	≥62 h/week	*p*-value
		4,161 (31.21%)	5,303 (39.77%)	3,869 (29.02%)	
Depressive symptoms, mean ± SD	5.78 ± 3.62	5.55 ± 3.61	5.71 ± 3.57	6.11 ± 3.70	0.051
Age, mean ± SD	40.06 ± 11.07	40.55 ± 11.07	39.40 ± 11.14	40.46 ± 10.93	0.420
Gender					<0.001
Male	57.47	50.68	57.93	64.13	
Female	42.53	49.32	42.07	35.87	
Marital status					<0.001
Unmarried	17.33	18.00	18.86	14.53	
Married	79.67	79.02	78.31	82.22	
Divorced or bereavement	3.00	2.98	2.83	3.26	
Education level					<0.001
Middle school and below	62.60	50.18	60.91	78.29	
High school/vocational school	16.60	15.26	18.76	15.07	
College and above	20.80	34.56	20.33	6.64	
Personal income, mean ± SD ^b^	6.92 ± 4.94	6.92 ± 5.01	7.30 ± 4.79	6.38 ± 5.00	0.001
Self-rated health					<0.001
Unhealthy	21.52	19.68	21.14	24.01	
Fair	45.64	47.87	45.28	43.73	
Healthy	32.84	32.44	33.58	32.26	
Chronic disease					0.036
No	89.19	88.18	89.76	89.51	
Yes	10.81	11.82	10.24	10.49	
Sleeping time weekday, mean ± SD	7.55 ± 1.32	7.52 ± 1.25	7.59 ± 1.23	7.53 ± 1.50	<0.001
Physical exercise, mean ± SD	2.15 ± 3.03	2.47 ± 3.12	2.16 ± 2.96	1.77 ± 2.98	0.001
Smoking					<0.001
No	64.73	70.63	64.02	59.34	
Yes	35.27	29.37	35.98	40.66	
Alcohol consumption					<0.001
No	83.00	85.94	82.71	80.25	
Yes	17.00	14.06	17.29	19.75	

^a^*p* values were calculated with the chi-square test or *F*-test using follow-up completed participants. ^b^Personal income was in logarithmic form.

### 3.2. The associations between overtime and depressive symptoms

Table 2 reports the associations between overtime and depressive symptoms. Premised on controlling socio-demographic characteristics, the overtime group (working hours ≥44 h/week) have an increased probability of depressive symptoms (OR = 1.12, 95%CI 1.04–1.20, Model 0), compared with those who reported working hours 30–44 h/week. What’s more, the longer the working time is, the more serious the depressive symptoms will be. The worsening of depressive symptoms is more pronounced in the heavy overtime group (working hours ≥62 h/week) (OR = 1.36, 95% CI 1.26–1.48). Health status indicators (self-rated health and chronic disease) are further added in Model 1. The results showed that both overtime group and heavy overtime group are prone to depressive symptoms (OR = 1.12, 95% CI 1.04–1.20; OR = 1.34, 95% CI 1.23–1.45), moreover, heavy overtime group are most likely to have depressive symptoms. Model 2 further adds health behaviors indicators (physical exercise, smoking, alcohol consumption and sleeping time weekday), and the results remain robust.

**Table 2:** Multivariable ordered logistic regression analysis examining the association between overtime and depressive symptoms.

Variables	Model 0			Model 1			Model 2		
	OR	95% CI	*p*	OR	95% CI	*p*	OR	95% CI	*p*
Working hours (ref: 30–44 h/week)									
44.1–61.9 h/week	1.12	1.04–1.20	0.002	1.12	1.04–1.20	0.003	1.11	1.04–1.20	0.003
≥62 h/week	1.36	1.26–1.48	<0.001	1.34	1.23–1.45	<0.001	1.32	1.22–1.44	<0.001
Age	0.99	0.99–0.99	<0.001	0.98	0.98–0.98	<0.001	0.98	0.98–0.98	<0.001
Gender (ref: male)	1.51	1.42–1.60	<0.001	1.43	1.35–1.52	<0.001	1.52	1.40–1.64	<0.001
Marital status (ref: unmarried)	.			.			.		
Married	0.94	0.85–1.03	0.199	0.90	0.82–0.99	0.035	0.89	0.81–0.98	0.021
Divorced or bereavement	2.25	1.84–2.76	<0.001	2.20	1.79–2.70	<0.001	2.15	1.75–2.63	<0.001
Education level (ref: middle school and below)	.			.			.		
High school/ vocational school	0.89	0.82–0.96	0.005	0.89	0.81–0.96	0.005	0.89	0.82–0.97	0.009
College and above	0.92	0.84–1.00	0.063	0.90	0.82–0.98	0.017	0.91	0.83–0.99	0.029
Personal income	0.99	0.98–0.99	<0.001	0.99	0.98–1.00	0.005	0.99	0.98–1.00	0.003
Self-rated health (ref: bad)				.			.		
Fair				0.58	0.53–0.63	<0.001	0.58	0.54–0.63	<0.001
Good				0.31	0.29–0.34	<0.001	0.32	0.29–0.35	<0.001
Chronic disease (ref: no)				1.52	1.37–1.68	<0.001	1.53	1.38–1.70	<0.001
Sleeping time weekday							0.96	0.93–0.98	<0.001
Physical exercise							0.99	0.98–0.99	0.003
Smoking (ref: no)							1.11	1.03–1.20	0.009
Alcohol consumption (ref: no)							1.01	0.93–1.10	0.738

### 3.3. The moderating effect of age, education level, and income level

We further test the moderators including age, education level, and income level, which could explain the differential associations between overtime and depressive symptoms. Specifically, we introduce the interaction term of overtime and these three variables to test the moderating effect. The measurement results are presented in Models 3–5 in Table 3.

**Table 3:** Regression analyses testing the moderating effects of age, personal income, education level.

	Model 3			Model 4			Model 5		
Variables	OR	95% CI	*p*	OR	95% CI	*p*	OR	95% CI	*p*
Overtime (ref: non)									
44.1–61.9 h/week	1.23	0.94–1.61	0.133	0.91	0.80–1.03	0.127	1.00	0.91–1.11	0.953
≥62 h/week	2.07	1.53–2.80	<0.001	1.02	0.89–1.16	0.791	1.21	1.10–1.34	<0.001
44.1–61.9 h/week *age	1.00	0.99–1.00	0.486						
≥62 h/week * age	0.99	0.98–1.00	0.003						
44.1–61.9 h/week * personal income				1.03	1.02–1.05	<0.001			
≥62 h/week * personal income				1.04	1.03–1.06	<0.001			
44.1–61.9 h/week * high school/vocational school							1.24	1.01–1.51	0.026
44.1–61.9 h/week * college and above							1.25	1.06–1.48	0.004
≥62 h/week * high school/vocational school							1.15	0.93–1.44	0.124
≥62 h/week * college and above							1.44	1.12–1.85	0.001
Age	0.99	0.98–0.99	<0.001	0.98	0.98–0.98	<0.001	0.98	0.98–0.98	<0.001
Personal income	0.99	0.98–1.00	0.004	0.97	0.95–0.98	<0.001	0.99	0.98–1.00	0.015
Education level (ref: middle school and below)									
High school/vocational school	0.89	0.82–0.97	0.01	0.91	0.84–0.99	0.033	0.78	0.66–0.91	0.002
College and above	0.93	0.85–1.01	0.089	0.96	0.88–1.05	0.363	0.78	0.69–0.88	<0.001

The results show that after controlling individual characteristic variables, health status variables, and health behavior variables, the interaction terms of age, personal income, education level variables and overtime work have significant impacts on an individual’s depressive symptoms (Table 3). The cross-term coefficient of age and heavy overtime (OR = 0.99, 95% CI 0.98–1.00) shows that the older the individuals are, the higher risk of depressive symptoms they will have because of heavy overtime. In Model 4, according to the cross-term coefficient of income variable, overtime variable, and heavy overtime variable (OR = 1.03, 95% CI 1.02–1.05; OR = 1.04, 95% CI 1.03–1.06, Model 4), the higher the income is, the more likely overtime work will lead to depressive symptoms. In Model 5, the cross-term of education level and heavy overtime shows that the higher the education level is, the more likely overtime will lead to depressive symptoms.

## 4. Discussion

By analyzing data from CPFS2018, we find a significant association between overtime and depressive symptoms. In addition, we also find that the association between overtime and depressive symptoms is heterogeneous by age, education level, and income level. Specifically, younger employees, employees with higher education levels, and employees with higher income levels are more likely to report depressive symptoms. These findings can help to explain why some existing studies have inconsistent results on the relationship between overtime and depressive symptoms. We believe that the differences in results from previous studies are likely to be influenced by the characteristics of participants. Specifically, age is one of the potential factors that influence the link between overtime and depressive symptoms (16). Younger employees are more likely to be affected by long working hours and exhibit symptoms of depression. The heterogeneity analysis of overtime work on depressive symptoms may also exist in groups with different educational and income levels (17, 18), which is worthy of further discussion.

Recent systematic reviews and meta-analyses suggest a positive relationship between overtime and depression (19, 20). The conclusions of this paper are consistent with the above studies. In addition, in this study, heavy overtime (working hours≧62 h/week) shows more obvious depressive symptoms than overtime (working hours≧44 h/week). This is consistent with the previous studies that the risk of mental illness is predominant among those who work the longest hours (11, 21, 22). The average working hours per week over 44 h and 62 h means that the average working hours per day are ≧7.3 h and ≧10.3 h after deducting the statutory holiday. Obviously, the time spent on maintaining basic human survival needs, such as sleeping and entertainment, are all part of employees’ non-working time. According to time substitution theory, working hours and non-working hours are negatively correlated. Extending employees’ working hours will shorten their non-working hours, resulting in having less time to relax or recover effectively (23, 24). For example, sleeping is an essential recovery activity for human beings, which has the effect of promoting recovery for both mental and physical strength. Lacking of sleep will hinder the effective recovery of employees and exacerbate the negative effects of mental exhaustion caused by long overtime work (25). Nakata (26) found that the combination of long working hours and short sleep was the greatest risk for depressive symptoms among full-time employees.

Besides, this study finds that multiple factors may moderate the relationship between overtime and depressive symptoms. One of the moderating factors is age. Most of the existing studies have introduced age as a control variable into the determining equation of depressive symptoms but ignored the moderating effect of age on the relationship between overtime work and depressive symptoms (7, 27). Our results suggest that when employees of different ages experience overtime, younger employees are more likely to develop depressive symptoms than the older group. Specially, younger employees are having less control over work and high work demands that make them work overtime. Previous studies have suggested that this may be related to the position of employees (4). Older employees are often promoted to managers based on their accumulated workability and experience, and they enjoy more autonomy in overtime work, so they are less prone to depression symptoms. We believe that the adjustment of age on the relationship between overtime and depressive symptoms may also be related to employees’ mental maturity and psychological tolerance. According to the personality development theory, older employees have more mature mental ability and psychological endurance to cope with stressful events such as working long hours and have less subjective distress to negative events (28). In addition, as the “Sandwich generation,” senior employees are the main breadwinners of the original family and the new family, and they need to take responsibility for caring for parents and bringing up children at the same time. According to psychologist Clark Hull’s drive theory, they are more likely to voluntarily work overtime driven by the need for more labor compensation (29). Therefore, we can speculate that younger employees, who generally experience fewer stages of personality development, have lower mental maturity and psychological endurance than older employees. Thus, when faced overtime work, young employees will have a stronger and an unbearable painful feeling due to their weak mental ability, and thus have a lower tolerance for overtime work, leading to more prone to depressive symptoms (16). In brief, older employees are less likely to develop depressive symptoms because working long hours can satisfy their need for financial compensation.

Moreover, we also found that the educational level of employees would moderate the relationship between overtime and depressive symptoms. However, it is inconsistent with the conclusion of previous studies that higher education has a negative moderating effect on the relationship between the two variables (18, 30). This study found that employees with higher education levels are more likely to develop depressive symptoms when working overtime. There is no doubt that in the same state of overtime, a higher level of education helps employees to have more direct remuneration and promotion opportunities and other work resources than those with a lower level of education (31). However, the material incentive for highly educated employees to work overtime have a diminishing marginal effect. Meanwhile, overtime squeezes out employees’ non-working time, which leads to an insufficiency of spare time for highly educated employees to enjoy their spiritual life even after meeting their material needs. And highly educated employees tend to have higher employment expectations, that is, a desire to enjoy life while pursuing their career (32). This contradiction between reality and ideal will further aggravate the painful feeling and ambivalence of highly educated employees when they work overtime, thus accelerating their mental consumption and making them more prone to depressive symptoms. However, the conflicts mentioned above are relatively less severe among less-educated employees, so the depressive symptoms of the low-educated group are relatively insignificant.

Finally, the result is found that income level significantly moderates the association between overtime and depressive symptoms. Some scholars believe that the income level of employees has a negative moderating effect (4). They find that high-income overtime employees can use their income to make up for the free time occupied by overtime so that they can get a better recovery (33). Therefore, they are less prone to depressive symptoms than low-income employees. However, our study draws an opposite conclusion that depressive symptoms are more pronounced in high-income employees who experience work overtime (32). This may be due to the prevalence of overtime culture in China. Additionally, unlike Western countries, China’s trade unions are internal departments of the enterprise and do not have enough bargaining power on behalf of workers (34).Therefore, workers’ rights and interests in working hours lack the protection of trade union organizations. This makes it difficult for high-income workers to refuse to work overtime after achieving relative freedom of wealth. Therefore, this may partly explain the higher likelihood of depressive symptoms among high-income employees who work overtime. In addition, lower-income employees are usually in basic positions with more physical labor and less work responsibility, while those with higher income are usually in key positions with more mental work and more work responsibility (35). Correspondingly, high-income employees need to put more effort into work, so their spirits are often in a high state of tension, resulting in serious mental internal friction. Meanwhile, mental recovery takes longer than physical recovery. Therefore, the mental burnout and aversion caused by overtime are more significant in high-income employees, which makes high-income employees more prone to depressive symptoms during overtime work.

## 5. Limitations and future research perspectives

Although this study guarantees scientific norms in quantitative research, there are still some limitations and deviations. First, the selected samples may suffer from participants who are working but may be on long-term leave from their work or have not worked for a long term. However, the limited information provided by the data makes it difficult for us to identify and exclude these sample. Second, this is a cross-sectional study, so we cannot make temporal inferences or capture dynamic evolution trends. Future studies can use longitudinal studies or experimental designs to corroborate this relationship. Third, overtime working hours were measured by self-report, which may cause recall bias. Follow-up studies can avoid this problem by obtaining real-time data on daily overtime work from company data or employees.

## 6. Conclusion

By analyzing data from CFPS2018, this study explores the association between overtime and depressive symptoms in employees from China, an Asian country where overtime is more typical. Ordered logistic regression results showed that working overtime increased the likelihood of depressive symptoms, and this effect remained significant after controlling for individual characteristics, health status, and health behavior variables. In addition, the moderating effect results show that age, education level, and income level moderate the association between overtime and depressive symptoms. Specifically, younger employees, employees with high education levels, and those with high income are more likely to be affected by the negative effects of overtime. It can be seen that to reduce the health burden brought by overtime work to employees, the government and enterprises should not only shorten the overtime working hours but to reduce the risk of depressive symptoms caused by the continuous overload of physical and mental consumption. In addition, the role of age, education level, and income level in regulating the relationship between overtime work and depressive symptoms should also receive more attention. Targeted mental health examinations and corresponding psychological interventions should be provided for employees who are more likely to develop depressive symptoms during overtime work, thereby reducing the possibility of the above-mentioned groups of employees developing depressive symptoms due to overtime work.

## Data availability statement

The original contributions presented in the study are included in the article/supplementary materials, further inquiries can be directed to the corresponding authors.

## Ethics statement

The 2018 Chinese Family Panel Studies (CFPS) data used in this study were approved by the Biomedical Ethics Committee of Peking University. The review lot number is unified: IRB00001052-14010.

## Author contributions

All authors contributed to the study conception and design. YL wrote the manuscript with support from PL. YL, PL, and ZL provided comments on the final draft and finished the final draft. XiaW provided critical feedback and helped shape the research. XinW performed the analytic calculations and performed the numerical simulations. LM was in charge of the revision. All authors discussed the results and contributed to the final manuscript.
